# In This Issue: Volume 116, Issue 7, July 2025

**DOI:** 10.1111/cas.70125

**Published:** 2025-07-01

## Dual Targeting of Aurora‐A and Bcl‐xL Synergistically Reshapes the Immune Microenvironment and Induces Apoptosis in Breast Cancer

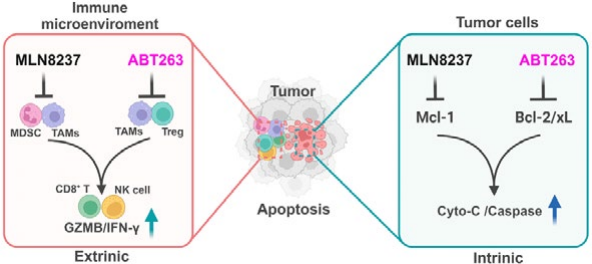

Triple‐negative breast cancer (TNBC) is one of the most aggressive forms of breast cancer that often spreads quickly and does not respond to many common treatments. Existing drugs can have limited effects when used alone and often cause serious side effects. To overcome these challenges, scientists are exploring whether combining different drugs at lower doses can offer better and safer treatment options.

In this issue, Liu et al. studied the effects of using two drugs—MLN8237 and ABT263—together to treat TNBC. MLN8237 blocks a protein called Aurora‐A that helps cancer cells divide, while ABT263 targets proteins like Bcl‐xL that help cancer cells avoid death. The researchers tested this drug combination in both cell cultures and mouse models of TNBC.

They found that the two drugs worked much better together than alone. In mice with a working immune system, the combination slowed tumor growth and reduced the spread of cancer to other parts of the body. In contrast, the treatment was less effective in mice without a functioning immune system, showing that the body's natural defenses play a major role in the drug's success.

Further tests revealed that the combination boosted the activity of immune cells that fight cancer, such as CD8^+^ T cells and natural killer (NK) cells. At the same time, it reduced the number of immunosuppressive immune cells (e.g., regulatory T cells and tumor‐associated macrophages). This change made the immune system more aggressive against the cancer and helped trigger a process called apoptosis, where cancer cells self‐destruct.

The treatment also caused cancer cells to die by shutting down their internal “survival signals.” Specifically, it blocked proteins that normally protect cancer cells from dying. Interestingly, the drugs did not stop cancer cells from growing but mainly encouraged them to die, and importantly, they did this without harming healthy tissues.

These findings highlight the potential of combining MLN8237 and ABT263 as a new approach to treating TNBC. By both targeting cancer cells and enhancing the immune system's ability to fight them, this strategy may offer a promising direction for developing more effective and gentler treatments in the future.

https://onlinelibrary.wiley.com/doi/full/10.1111/cas.70072

## Steroid‐Modulated Transcription Synergistically Forms DNA Double‐Strand Breaks With Topoisomerase II Inhibitor

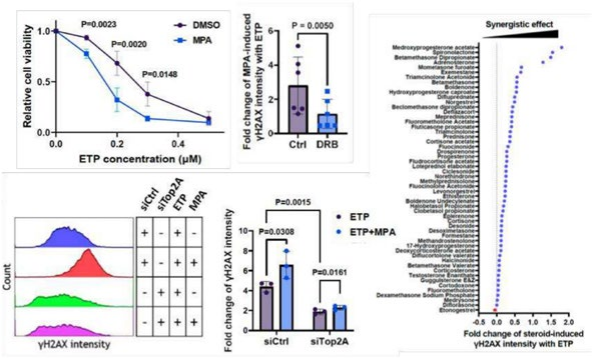

Chemotherapy as a treatment approach makes use of potent drugs to damage the DNA of cancer cells, restricting their growth and survival. But over time, cancer cells can become resistant to these drugs, or the drugs might lose their potency to achieve a lasting effect. For example, etoposide (ETP), a commonly used chemotherapy drug, works by inducing breaks in cancer cell DNA, effectively halting their growth. While ETP generally works well, it does not always have the same effect on every patient, and stronger doses can increase side effects. That is why researchers are working on innovative approaches to enhance the effectiveness of drugs like ETP without increasing their harmful side effects.

Interestingly, recent findings have shown that medroxyprogesterone acetate (MPA)—a steroid commonly used for birth control and hormone therapy—can make ETP work significantly better.

Now, researchers discovered that MPA, when combined with ETP, dramatically increases the damage to cancer cell DNA. In this study conducted by Zhao and colleagues, the team grew human cancer cells, including those from cervical cancer, lung adenocarcinoma, and colorectal cancer and treated them with ETP and various hormone‐based drugs. They found that MPA promotes the formation of lethal DNA damage from a genomically unstable state in which the topoisomerase II (Top2) is trapped on DNA by ETP. This unique interaction makes it harder for cancer cells to survive and could lead to improved treatment outcomes. Interestingly, this effect was not seen when MPA was combined with other drugs, indicating a novel mechanism of action for this drug combination, unlike conventional strategies that focus solely on inhibiting DNA repair.

The researchers further found promising results on combining many other similar steroids with ETP. These effects, however, could not be consistently replicated in animal models because MPA did not effectively reach the tumors. Even so, the results of this study are encouraging and suggest that new steroid alternatives with improved delivery properties can be explored further.

Using hormones synergistically to boost the ability of existing drugs to target DNA precisely, this study offers a novel strategy for cancer sensitization to the drugs. By making existing cancer drugs more efficient, these findings open up new possibilities in the field of chemotherapy.

https://onlinelibrary.wiley.com/doi/full/10.1111/cas.70081

## Evans Blue Acts as a Selective Inhibitor of CaMKII‐α to Impede the Progression of TCL Identified by HTS

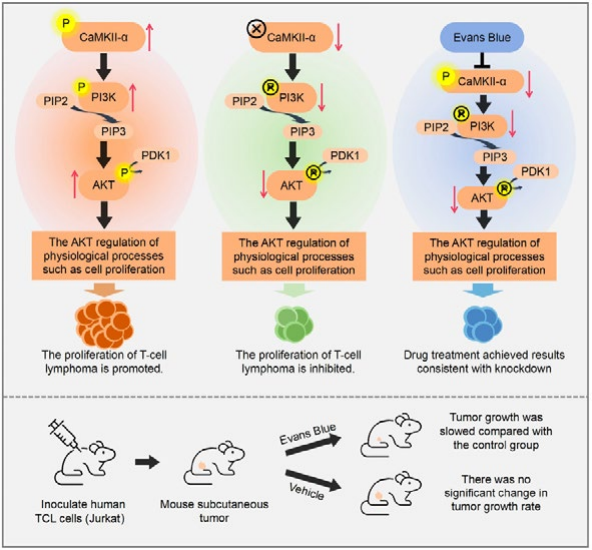

T‐cell lymphoma (TCL) is an aggressive form of cancer that affects T‐cells, a type of white blood cell crucial for defending the body against infections and diseases. Treating TCL presents a major challenge, as the disease often resists standard therapies such as chemotherapy and radiation, which can cause serious side effects and may lose effectiveness over time.

To address this, researchers explored the potential of the protein Ca^2+^/calmodulin‐dependent protein kinase II alpha (CaMKII‐α) as a possible therapeutic target for the treatment of TCL. While CaMKII‐α is primarily expressed in the nervous system, it belongs to a critical class of proteins that play an important role in regulating cellular processes such as cell growth, survival, and gene expression.

The researchers found that CaMKII‐α is overexpressed in TCL cells. By using small interfering RNA (siRNA) they removed the gene for CaMKII‐α, and were able to significantly slow down the growth of these cancer cells. Their findings also suggested that CaMKII‐α may promote cancer progression by activating the PI3K‐AKT signaling pathway, a key driver of cell growth and survival commonly linked to many types of cancer. Furthermore, the team discovered Evans Blue—a molecule originally used as a dye—showed promise as a candidate for inhibiting CaMKII‐α activity.

Although Evans blue has traditionally been used for staining, recent studies have shown that it has potential applications in medicine, including its ability to bind to plasma proteins and assist in the diagnosis of cerebrovascular diseases. Laboratory experiments showed that Evans Blue had strong tumor‐suppressive effects at very low doses, potentially via regulation of the CaMKII‐α‐PI3K‐AKT pathway.

These findings suggest that Evans Blue, a drug approved by the United States Food and Drug Administration, could be used as a selective CaMKII‐α inhibitor to treat TCL, representing a promising target for therapeutic strategies. This study underscores the need for continued research to validate the role of CaMKII‐α in TCL, including animal studies, and to explore additional mechanisms by which Evans Blue may inhibit cancer progression.

https://onlinelibrary.wiley.com/doi/full/10.1111/cas.70051

